# Supplementary material for: Development and Stability of a New Formulation of Pentobarbital Suppositories for Paediatric Procedural Sedation
Source: Pharmaceutics. 2023 Feb 24;15(3):755. doi: 10.3390/pharmaceutics15030755 (PMC10055724; doi:10.3390/pharmaceutics15030755)
Supplement: Supplementary file 1 [file pharmaceutics-15-00755-s001.zip › Supplementary data file S1.pdf]

Article

Development and stability of a new formulation of pentobarbital suppositories for pediatric procedural sedation

Matthieu Lebrat[1], Yassine Bouattour[2*], Coralie Gaudet[1], Mouloud Yessaad[1], Mireille Jouannet[1], Mathieu Wasiak[1], Imen Dhifallah[3], Eric Beyssac[3], Ghislain Garrait[3], Philip Chennell[2*], Valérie Sautou[2]

[1] CHU Clermont-Ferrand, Pôle Pharmacie, F-63003 Clermont-Ferrand, France
[2] Université Clermont Auvergne, CHU Clermont Ferrand, Clermont Auvergne INP, CNRS, ICCF, F-63000 Clermont-Ferrand, France
[3] Université Clermont-Auvergne, UFR Pharmacie, UMR MEDIS, Clermont-Ferrand F-63001, France
* Correspondence: pchennell@chu-clermontferrand.fr (P.C.) and ybouattour@chu-clermontferrand.fr (Y.B.)

SUPPLEMENTARY DATA FILE S1.

Determination of oleic acid amounts in formulation 2

For F2 formula, amounts of oleic acid was determined experimentally by adding it drop by drop at 80°C to a pentobarbital-Witepsol® W25 preparation at a ratio corresponding to F1 formula of each dosage, until getting a clear solution. The found values were considered to be the minimum amount of Oleic acid (OA) to be in the formula per suppository (**Table S1**).

Table S1. Minimum amount of oleic acid per suppository for each dosage

Amount of Pentobarbital per suppository	30 mg	40 mg	50 mg	60 mg
Minimum amount of Oleic acid per suppository	0.223 g	0.253 g	0.280 g	0.304 g

The displacement factor (f) of oleic acid in Witepsol®W25 base can also be determined by dividing the density of Witepsol® W25 (0.96) by the density of oleic acid (0.895), which is equal to 1.07.

Then, the amount of Witepsol® W25 in F2 formula was defined using the formula W = S – (OA*f) with W = Witepsol® W25 weight to be added per suppository and S = total weight of one suppository (equal to 1.1g).

Validation of the extraction method

The extraction method of pentobarbital from suppositories were performed for three consecutive days. Each day, 6 suppositories of 30 mg pentobarbital of F1 and F2 formulations were prepared individually. After 24 hours of cooling, each suppository were solubilized in 10 mL of octanol at 50°C and then transferred in an extraction flask with 20 mL of NaOH solution at 0.1M. After 24 hours of extraction, 100 µL of the lower aqueous solution were diluted in 10 mL of sterile water and then quantified using UV-visible spectrophotometry. Suppositories without pentobarbital of each formulation were also analyzed to verify that no matrix effect were present. The results are presented in Table S2:

Table S2. Extraction yield for both formulations (n = 18, values expressed as mean ± standard deviation)

Formulation 1		Formulation 2	
Suppositories content (mg)	Extraction yield (%)	Suppositories content (mg)	Extraction yield (%)
29.91 ± 0.53	99.20 ± 3.39	30.37 ± 0.32	101.03 ± 2.22

Pentobarbital peak purity assessment after 41 weeks of storage.

Peak purity was of 100% for the totally of the pentobarbital peak (see Figure S1 for representative peak purity assessments).

(A) (B)

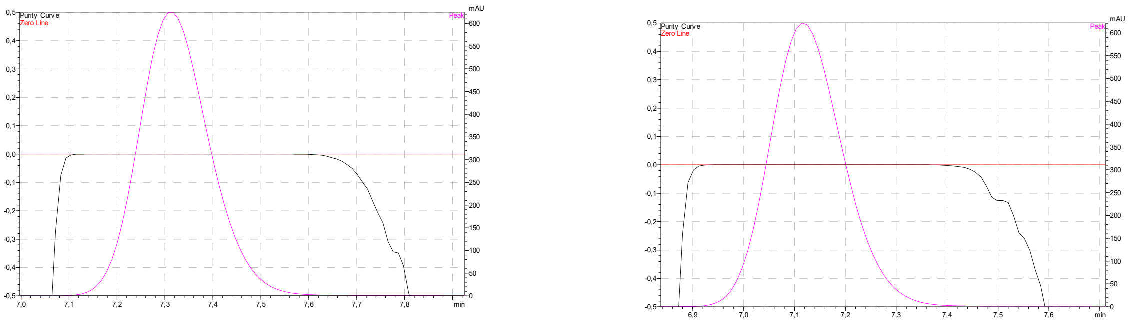

Figure S1. Peak purity assessment of (A) 30 mg pentobarbital suppositories and (B) 60 mg pentobarbital suppositories after 41 weeks of storage.
